# Association of the serum sialic acid-to-albumin ratio with prostate cancer and bone metastasis: a retrospective cross-sectional study

**DOI:** 10.7717/peerj.21824

**Published:** 2026-10-27

**Authors:** Yu Ji, Chunhui Guo, Yuxuan Zhao, Li Yi, Renxuan Liu, Laiyuan Qiu, Gangli Gu, Dawei Li

**Affiliations:** 1 Department of Urology, Qilu Hospital of Shandong University, Jinan, Shandong, China; 2 Cheeloo College of Medicine, Shandong University, Jinan, Shandong, China; 3 Department of Oncology, Binzhou Central Hospital, Binzhou, Shandong, China

**Keywords:** Prostate cancer, Benign prostatic hyperplasia, Bone metastasis, Serum sialic acid, Serum albumin, Prevention

## Abstract

**Background:**

Prostate cancer (PCa) is a growing global health concern, with bone metastasis significantly worsening outcomes. Current diagnostics such as prostate-specific antigen (PSA) lack specificity, therefore, this study aimed to evaluate the clinical utility of the serum sialic acid-to-albumin ratio (SAAR) in assisting the identification of PCa and assessing bone metastasis status.

**Materials and Methods:**

This study retrospectively collected the clinical data form patients diagnosed with PCa and benign prostatic hyperplasia (BPH) at Qilu Hospital of Shandong University from August 2014 to February 2022. SAAR levels were compared between the two groups, and receiver operating characteristic (ROC) curve analysis was performed to assess its discriminative capability. The bone metastasis cohort included 254 patients with PCa whose bone metastasis status was clearly determined, randomly split into training and testing sets. Least absolute shrinkage and selection operator regression (LASSO) was used for variable selection in the training set, followed by multivariable logistic regression to develop a model for assisting in the identification of bone metastasis. Model performance was evaluated using the ROC curve, calibration curve, and decision curve analysis (DCA). To evaluate the incremental value of SAAR, model area under curves (AUCs) were compared using the DeLong test, alongside the calculation of net reclassification improvement (NRI) and integrated discrimination improvement (IDI).

**Results:**

SAAR levels were higher in PCa patients compared to BPH patients, although the ability to differentiate PCa from BPH was limited. Among patients whose bone metastasis status was clearly determined, SAAR levels were significantly higher in those with bone metastasis. Multivariable logistic regression suggested that SAAR may be independently associated with bone metastasis. Further comparisons across multiple models showed that incorporating SAAR into the baseline model of PSA and Gleason score modestly improved discrimination and risk reclassification. The clinical model incorporating SAAR, lactate dehydrogenase (LDH), PSA, and pathological grade demonstrated favorable performance.

**Conclusion:**

SAAR is associated with PCa and the presence of bone metastasis. Its ability to differentiate PCa from BPH is limited, but it may provide adjunctive information for identifying bone metastasis. The proposed model show s acceptable internal performance and requires external validation before clinical use.

## Introduction

Prostate cancer (PCa) represents a substantial global health burden and ranks second in cancer incidence among men. In 2022, PCa accounted for 1,466,680 new cases globally (7.3% of all newly diagnosed cancers), with 396,792 deaths (Bray et al., 2024). Incidence continues to increase, with marked geographic variations in both incidence and mortality. Higher rates are reported in the Caribbean, Australia, and New Zealand, whereas lower rates are found in parts of Asia and North Africa (Rawla, 2019). The risk is significantly related to factors such as age, race, genetic background, and lifestyle. Of these, advancing age remains the predominant factor, with incidence increasing after 65 years (Pernar et al., 2018).

Metastasis is a key factor associated with poor prognosis in PCa. Bone is the predominant site of metastatic dissemination in PCa, with skeletal involvement affecting up to 70% of patients with advanced disease (Lin, Mokgautsi & Liu, 2020). Bone metastasis induces severe skeletal-related events (osteolytic pain, bone lesions, and neurologic compromise), which reduce the quality of life and shorten survival (Mollica et al., 2021). In terms of surgical oncology, the presence or high suspicion of bone metastasis can directly affect treatment strategies. Therefore, the early identification of metastasis in PCa is essential to enhance patient outcomes. Current clinical diagnosis system combines prostate-specific antigen (PSA) screening with imaging and biopsy (Mottet et al., 2021). While PSA screening has greatly improved early detection, low specificity and sensitivity have led to overdiagnosis and overtreatment of PCa (Schröder et al., 2009). Thus, the current major research focus is to combine PSA with more reliable biomarkers to improve the accuracy of PCa diagnosis and risk stratification.

Serum biomarkers have been particularly scrutinized in the context of PCa research over the years. Sialic acid (SA), an oxygenated sugar molecule, is involved in cell recognition and signal transduction and has been implicated in tumor initiation, progression, and metastasis. SA expression is markedly upregulated on prostate tumor cells, which may promote tumor growth and bone metastasis (Hodgson et al., 2024). Serum albumin (ALB) is a key indicator of nutritional status and the systemic inflammatory response, and may reflect systemic inflammation or cancer-associated cachexia (Kühn et al., 2017). SA has become a promising tumor biomarker in nasopharyngeal, breast, gynecologic, and prostate cancer, yet its specificity can be affected by non-tumor conditions such as infection and inflammation (Chen et al., 2024; Lomax-Browne et al., 2019; Berghuis et al., 2022; Sun et al., 2024). By incorporating ALB, the serum sialic acid-to-albumin ratio (SAAR) may overcome non-tumor factors interference and provide more robust information for the assessment of PCa and bone metastasis.

However, research on SAAR in PCa and bone metastasis remains relatively scarce. In light of this, we evaluated the association of SAAR with PCa and bone metastasis using a retrospective cross-sectional dataset. We also examined whether SAAR added predictive information to combinations of PSA and Gleason score, and developed a multivariable model for identifying bone metastasis in patients with PCa.

## Materials & Methods

### Research participant curation

The present study collected urological records of cases newly diagnosed with PCa or benign prostatic hyperplasia (BPH) at Qilu Hospital of Shandong University from August 2014 to February 2022. The cohort comprised 691 patients, including 506 PCa and 185 BPH cases.

### Ethics statement

The study was approved by the Medical Ethics Committee of Qilu Hospital of Shandong University (Approval No. KYLL-202208-044). The requirement for informed consent was waived owing to the retrospective nature of the study. All patient information was kept strictly confidential.

### Inclusion criteria

(1) Diagnosis and time range: PCa group, patients pathologically diagnosed with prostate adenocarcinoma between August 2014 and February 2022, including localized and metastatic PCa; BPH group, patients diagnosed with BPH during the same period at the same hospital.

(2) Age range: Male patients aged 45–85 years.

(3) Clear bone metastasis status: Patients with or without bone metastasis confirmed by imaging studies such as emission computed tomography (ECT).

(4) Complete key clinical data: baseline clinical data (age, body mass index (BMI), comorbidities, *etc.*), complete pre-treatment blood test records (including SA, ALB, PSA, *etc.*), pathological reports (including Gleason score and TNM staging), and imaging reports.

(5) Treatment-naive or no prior systemic treatment: Patients who were treatment-naive or had not received systemic treatment (radiotherapy, chemotherapy, immunotherapy, or androgen deprivation therapy) to avoid the impact of treatment on SAAR.

(6) Other: no history of malignancy other than PCa. Patients with hematological diseases (such as anemia, leukemia, and myelodysplastic syndrome) were excluded to avoid interference with blood markers.

### Exclusion criteria

(1) Other malignancies: Patients with other primary malignancies (such as lung cancer, and colorectal cancer) or a history of metastatic tumors.

(2) Inflammatory or infectious conditions, including but not limited to chronic prostatitis, urinary tract infections, or systemic inflammatory disorders, as verified through clinical or pathological evaluation.

(3) History of prostate surgery: Patients with any history of prostate surgery prior to biopsy were excluded.

(4) Non-adenocarcinoma prostate malignancies: pathological diagnosis of non-adenocarcinoma types (such as urothelial carcinoma, sarcomatoid carcinoma).

(5) Drug interference: Patients who had used coagulants (such as vitamin K) or anticoagulants (such as warfarin and rivaroxaban) within the past two weeks or who had a long-term use of glucocorticoids or immunosuppressants.

(6) Incomplete records: Exclusion of patients with incomplete clinical data.

(7) Severe systemic diseases: severe liver or kidney dysfunction.

### Data collection

Patient electronic medical records were retrieved from the Qilu Hospital database, and relevant clinical data were collected, including age, smoking history, PSA, SA, ALB, fibrinogen (FIB), and pathological features (including pathological stage and grade). Pathological T staging followed the TNM criteria, whereas pathological grading was based on the Gleason score system. The patients were divided into high-grade (Gleason score >7) and low-grade (Gleason score ≤ 7) groups.

### Laboratory measurements

Fasting venous blood samples were collected before treatment. Serum levels of lactate dehydrogenase (LDH), α-L-fucosidase (AFU), PSA, SA, and ALB were measured in the hospital laboratory using standardized procedures. The reference ranges for blood test indicators in our hospital laboratory were as follows: LDH 120.00–230.00 U/L; FIB 2.0–4.00 g/L; AFU < 40.00 U/L; PSA < 4.00 ng/mL; SA 45.60–75.40 mg/dL; ALB 40.00–55.50 g/L.

### Assessment of bone metastasis

Bone metastasis status was primarily evaluated based on imaging reports, predominantly ECT, along with other available clinical imaging. Among the 506 patients with PCa, 79 individuals had confirmed bone metastasis, 175 individuals had no evidence of bone metastasis, 92 individuals had findings considered suspicious, and 160 individuals did not undergo ECT. The primary analyses and model construction for bone metastasis were restricted to the 254 patients with definitive bone metastasis status. To minimize potential outcome misclassification, patients with suspicious findings and those without ECT examination were excluded from these analyses.

### Statistical analysis

SAAR was calculated by dividing SA by ALB. Categorical variables were expressed using frequency (percentage) and compared using Pearson’s chi-squared test or Fisher’s exact test. The Kolmogorov–Smirnov (K-S) test was applied to evaluate the normal distribution, and the Quantile-Quantile (Q-Q) plot was used for additional verification. Because the continuous variables were non-normally distributed, they were described using median (interquartile range) and compared between groups using the Mann–Whitney U test. After evaluating the missing values for each variable, we found that the proportion of missing data was negligible. Therefore, no statistical imputation was performed, and a complete-case analysis approach was adopted.

Potential influencing factors and the association between individual variables and PCa were initially evaluated using univariable and multivariable logistic regression. Associations were estimated using odds ratios (OR) with 95% confidence intervals (CI). Variables with *P* < 0.05 in the univariable logistic regression were entered into the multivariable analysis. The optimal decision threshold for SAAR was established by maximizing the Youden index (sensitivity + specificity − 1) through receiver operating characteristic (ROC) curve analysis, with the area under the curve (AUC) calculated to assess overall discrimination.

To assess the incremental value of SAAR, a series of multivariable logistic regression models were sequentially constructed using the forced-entry method. The DeLong test was applied to evaluate whether the differences in the AUCs were statistically significant. Additionally, both categorical and continuous net reclassification improvement (NRI), along with the integrated discrimination improvement (IDI), were calculated to quantify the incremental value of SAAR.

To develop the model for identifying bone metastasis, 254 patients with definitive bone metastasis status were stratified and randomly split, with 80% assigned to the training set and 20% to the testing set. A fixed random seed was used to guarantee reproducibility. Subsequently, Least Absolute Shrinkage and Selection Operator (LASSO) regression with 10-fold cross-validation was performed within the training set for variable screening. Candidate predictors with non-zero coefficients at the 1-standard error threshold (*λ*_1se_) were selected and further incorporated into a multivariable logistic regression model. The resulting model was translated into a clinical nomogram, followed by assessment through ROC curves, calibration curves, and decision curve analysis (DCA). Finally, model performance was comprehensively evaluated using various metrics, including the AUC, accuracy, sensitivity, specificity, Brier score, and F1 score.

Statistical analyses were conducted using IBM SPSS Statistics (version 27.0; IBM Corp., Armonk, NY, USA) and R software, with two-sided *P* values < 0.05 denoting statistical significance.

## Results

### Clinical characteristics of BPH and PCa

This study included 506 patients with PCa and 185 with BPH. The pretreatment profiles of the two groups were as follows (Table 1). Compared to the BPH group, the PCa group exhibited a markedly elevated SAAR level (*P* < 0.001) (Fig. 1A). PSA levels differed significantly between the groups, with markedly elevated values in the PCa group than in the BPH group (*P* < 0.001). Moreover, LDH and FIB levels were increased in the PCa group, which is consistent with previous research findings (Mori et al., 2019; Yu, Yuan & Chen, 2023; Lei et al., 2023).

**Table 1:** Clinical characteristics of patients with BPH and PCa.

**Parameters**	**BPH**	**PCa**	***P***
**Patients**	185 (26.8%)	506 (73.2%)	
**Age**	68.00 (62.00, 74.00)	70.00 (64.00, 76.00)	0.006^*^
**LDH**	188.00 (167.00, 213.50)	194.00 (174.25, 225.00)	0.008^*^
**AFU**	17.00 (13.00, 21.00)	17.00 (13.00, 22.00)	0.562^*^
**FIB**	2.91 (2.56, 3.37)	3.19 (2.74, 3.71)	<0.001^*^
**PSA**	5.74 (2.73, 10.05)	40.75 (12.67, 134.30)	<0.001^*^
**SAAR**	1.22 (1.14, 1.39)	1.32 (1.18, 1.60)	<0.001^*^

**Notes.**

BPH benign prostatic hyperplasia PCa prostate cancer LDH lactate dehydrogenase AFU α-L-fucosidase FIB fibrinogen PSA prostate-specific antigen SAAR serum sialic acid-to-albumin ratio

Skewed data are represented by the median (Q1, Q3), and categorical data are represented by frequency (percentage, %).

* Mann–Whitney U test; *P* < 0.05 is considered as statistically significant.

**Figure 1:**
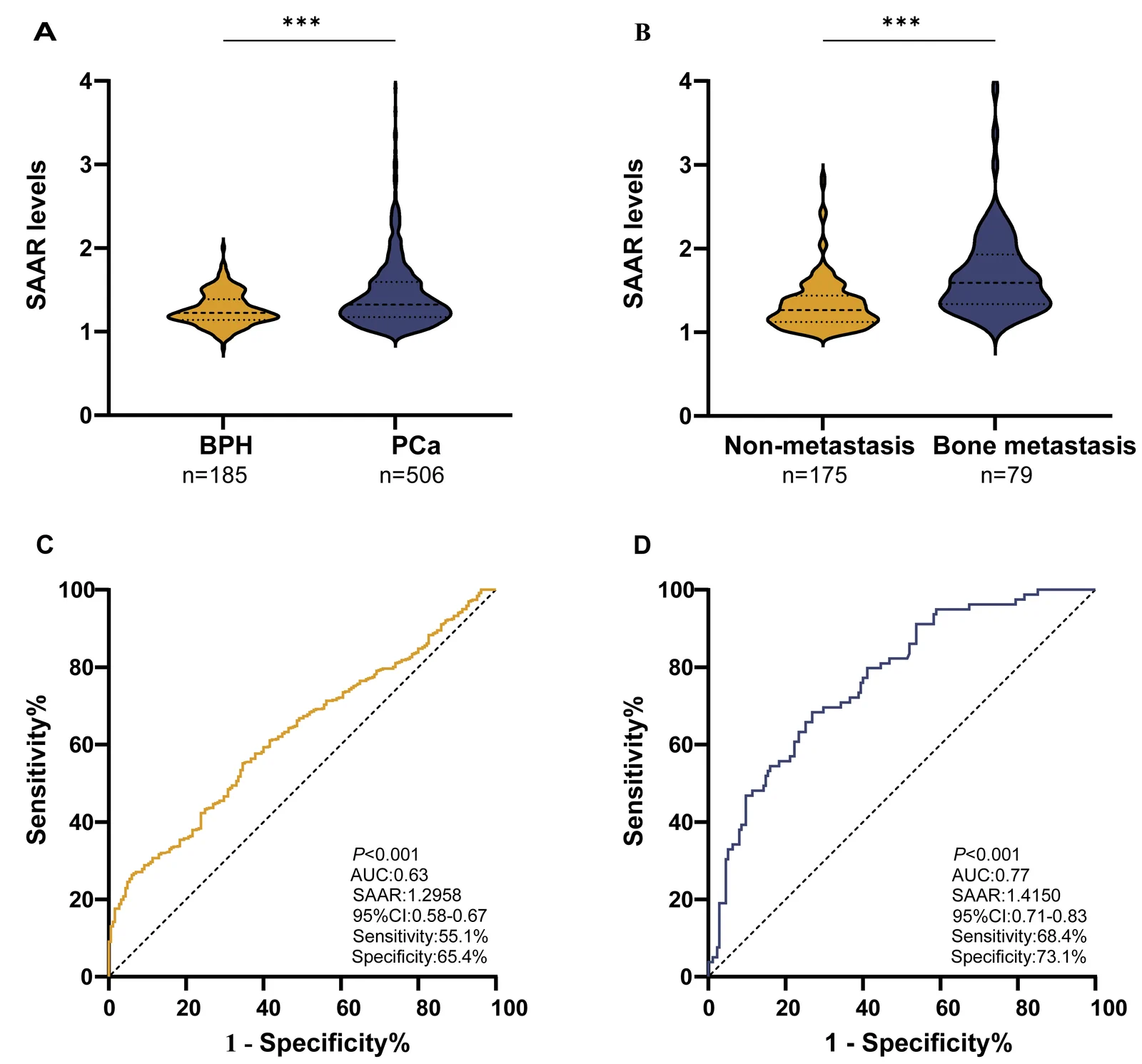
Distribution and ROC curve analysis of the serum sialic acid-to-albumin ratio in prostate cancer and bone metastasis. (A) Distribution of SAAR levels in patients with BPH and PCa. (B) Distribution of SAAR levels in patients with PCa according to definitive bone metastasis status. Among the 506 patients with PCa, 79 patients had confirmed bone metastasis, 175 patients had no evidence of bone metastasis, 92 patients had suspicious imaging findings, and 160 patients did not undergo ECT examination. The analysis in this panel was restricted to the 254 patients with definitive bone metastasis status (79 with bone metastasis and 175 without bone metastasis). (C) ROC curve of SAAR for differentiating PCa from BPH. The AUC was 0.63 (95% CI [0.58–0.67]). At the cutoff value of 1.2958, the sensitivity and specificity were 55.1% and 65.4%, respectively. (D) ROC curve of SAAR for identifying bone metastasis in patients with PCa whose bone metastasis status was definitively determined. The AUC was 0.77 (95% CI [0.71–0.83]). At the cutoff value of 1.4150, the sensitivity and specificity were 68.4% and 73.1%, respectively. SAAR levels were non-normally distributed and are presented as the median and interquartile range. Differences between groups were assessed using the Mann–Whitney U test. ***P < 0.001. SAAR, serum sialic acid-to-albumin ratio; PCa, prostate cancer; BPH, benign prostatic hyperplasia; ROC, receiver operating characteristic; AUC, area under the curve; ECT, emission computed tomography.

### Association between SAAR levels and clinical pathological parameters in BPH patients

In 185 BPH cases, SAAR levels were analyzed for association with clinicopathological characteristics (Table S1). Patients with elevated LDH (*P* = 0.038) or FIB (*P* < 0.001) exhibited markedly higher SAAR levels, suggesting that SAAR may be related to systemic inflammation or coagulation status. However, statistical analysis showed no significant differences in SAAR levels when stratified by age, smoking history, AFU, or PSA level (*P* > 0.05). These results indicate that changes in LDH and FIB levels may be associated with SAAR levels, while other clinical parameters may have a limited impact.

### Association between SAAR levels and clinical pathological parameters in PCa patients

Further analysis of the relationship between SAAR levels and clinical characteristics in 506 patients with PCa showed that SAAR levels were significantly associated with smoking history, LDH, FIB, PSA, and ECT results, but not with age or AFU levels (Table S1). Patients with elevated PSA levels showed significantly increased SAAR levels (1.41, 1.23–1.72) compared to those with lower PSA levels (1.26, 1.14–1.45) (*P* < 0.001), suggesting that SAAR levels may be associated with the biological activity or progression of PCa. For Gleason scores, the high-grade group showed SAAR levels substantially exceeding those of the low-grade group (*P* = 0.001). Although SAAR levels tended to be higher in pT3-4 cases relative to pT1-2 cases, the between-group difference was not statistically significant (*P* = 0.319).

Among the ECT results, 79 patients had bone metastasis, 175 patients had no bone metastasis, 92 patients had suspected bone metastasis, and 160 patients did not undergo ECT examination. The 254 patients with definitive bone metastasis status were retained for the bone-metastasis analyses. In this subset, compared with patients without bone metastasis, those with bone metastasis had significantly higher levels of LDH, AFU, FIB, PSA (all *P* < 0.05). The bone metastasis cohort showed the highest SAAR levels (1.59, 1.34–1.93). Specifically, patients with bone metastasis demonstrated significantly elevated SAAR levels compared to non-bone metastasis cases (1.26, 1.12–1.44) (Fig. 1B). A higher proportion of patients in the bone metastasis group had a high pathological grade (GS > 7) than among those without bone metastasis (*P* < 0.001). No significant between-group differences were observed in age or smoking history (*P* > 0.05) (Table 2).

**Table 2:** Baseline clinical characteristics of PCa patients with and without bone metastasis.

**Variables**	**Total (*n* = 254)**	**Non-bone metastasis (*n* = 175)**	**Bone metastasis (*n* = 79)**	***P***
**Age**	70.00 (64.00, 75.00)	71.00 (64.00, 75.00)	69.00 (64.00, 76.00)	0.564^*^
**LDH**	194.00 (173.00, 224.00)	186.00 (169.00, 211.00)	211.00 (186.50, 266.00)	<0.001^*^
**AFU**	17.00 (14.00, 23.00)	17.00 (13.00, 22.00)	19.00 (15.00, 24.00)	0.048^*^
**FIB**	3.15 (2.74, 3.64)	3.02 (2.66, 3.41)	3.64 (2.99, 4.34)	<0.001^*^
**PSA**	54.38 (16.13, 173.01)	31.92 (11.64, 83.00)	205.00 (64.64, 489.10)	<0.001^*^
**SAAR**	1.34 (1.17, 1.60)	1.26 (1.13, 1.43)	1.59 (1.34, 1.91)	<0.001^*^
**Smoking history** ^c^				0.228^a^
No	161 (63.64)	115 (66.09)	46 (58.23)	
Yes	92 (36.36)	59 (33.91)	33 (41.77)	
**Pathological grade** ^b^				<0.001^a^
Low (≤7)	107 (42.46)	88 (50.87)	19 (24.05)	
High (>7)	145 (57.54)	85 (49.13)	60 (75.95)	

**Notes.**

PCa prostate cancer LDH lactate dehydrogenase AFU α-L-fucosidase FIB fibrinogen PSA prostate-specific antigen SAAR serum sialic acid-to-albumin ratio

Pathological grades were classified into low-grade and high-grade groups based on the Gleason score. Skewed data are represented by the median (Q1, Q3), and categorical data are represented by frequency (percentage, %).

* Mann–Whitney U test.

a chi-square test; *P* < 0.05 is considered as statistically significant.

Only patients with definite bone metastasis status were included in this analysis, including 79 patients with bone metastasis and 175 patients without bone metastasis. Patients with suspected bone metastasis or without ECT examination were excluded from this comparison to minimize potential misclassification bias.

c Missing value for smoking history, *n* = 1, from the non-bone metastasis group.

b missing values for Pathological grade, *n* = 2, from the non-bone metastasis group. Missing values were excluded from the corresponding statistical tests, and percentages were calculated based on available data.

### Univariable and multivariable logistic regression models for PCa

Logistic regression models were employed to further assess the association of SAAR levels with PCa (Table 3). In the univariable analysis, SAAR showed a significant association with PCa (OR = 7.341, *P* < 0.001), indicating its potential importance in the diagnosis of PCa. PSA, as a traditional marker, also showed significant predictive ability (OR = 1.135, *P* < 0.001). Subsequently, age, LDH, FIB, PSA and SAAR levels were gradually incorporated into the multivariable regression model. LDH and FIB levels lost statistical significance in the multivariable analysis. The results indicated that, after adjusting for other variables, PSA and SAAR levels remained independently associated with PCa (PSA: OR = 1.134, *P* < 0.001; SAAR: OR = 2.655, *P* = 0.031).

**Table 3:** Univariable and multivariable regression analysis of variables associated with BPH *vs* PCa and PCa *vs* bone metastasis.

**Variables**	**Univariable regression** **OR (95% CI)**	***P***	**Multivariable regression** **OR (95% CI)**	***P***
**BPH *vs* PCa**				
Age	1.028 (1.008–1.049)	0.006	1.010 (0.985–1.036)	0.444
Smoking history	1.143 (0.803–1.629)	0.458	–	–
LDH	1.007 (1.003–1.011)	<0.001	1.002 (0.998–1.006)	0.368
AFU	1.009 (0.984–1.035)	0.481	–	–
FIB	1.718 (1.353–2.180)	<0.001	1.053 (0.760–1.460)	0.755
PSA	1.135 (1.101–1.170)	<0.001	1.134 (1.100–1.170)	<0.001
SAAR	7.341 (3.560–15.137)	<0.001	2.655 (1.092–6.455)	0.031
**PCa *vs* Bone metastasis**				
Age	0.991 (0.958–1.025)	0.610	–	–
Smoking history	1.398 (0.810–2.415)	0.229	–	–
LDH	1.013 (1.007–1.019)	<0.001	1.008 (1.002–1.014)	0.008
AFU	1.032 (0.994–1.071)	0.096	–	–
FIB	1.586 (1.220–2.062)	<0.001	0.997 (0.756–1.314)	0.982
PSA	1.006 (1.004–1.008)	<0.001	1.005 (1.003–1.007)	<0.001
Pathological grade (GS ≤7 *vs* >7)	3.269 (1.802–5.933)	<0.001	2.555 (1.224–5.334)	0.013
SAAR	11.937 (4.934–28.881)	<0.001	3.547 (1.139–11.039)	0.029

**Notes.**

BPH benign prostatic hyperplasia PCa prostate cancer LDH lactate dehydrogenase AFU α-L-fucosidase FIB fibrinogen PSA prostate-specific antigen SAAR serum sialic acid-to-albumin ratio OR odds ratio CI confidence interval

Pathological grades were classified into low-grade and high-grade groups based on the Gleason score. *P* < 0.05 is considered as statistically significant.

### Univariable and multivariable logistic regression models for bone metastasis in PCa

The association between SAAR levels and bone metastasis in patients with PCa was evaluated by establishing a logistic regression model (Table 3). Similarly, in both univariable and multivariable logistic regression analyses, SAAR remained significantly associated with bone metastasis, indicating that SAAR may be independently associated with the presence of bone metastasis in patients with PCa (univariable analysis: OR = 11.937, *P* < 0.001; multivariable analysis: OR = 3.524, *P* = 0.012). Furthermore, LDH, Gleason score and PSA levels were also independently associated with bone metastasis in the multivariable analysis.

### ROC curve analysis of SAAR levels in PCa diagnosis

To further describe the diagnostic capability of SAAR for PCa, an ROC curve was plotted (Fig. 1C). The optimal SAAR cutoff for diagnosing PCa was determined to be 1.2958, and SAAR levels above this threshold were significantly associated with PCa (*P* < 0.001). The AUC was 0.63, with a 95% CI [0.58–0.67]. Sensitivity, specificity and the positive predictive value (PPV) were 55.10%, 65.40% and 81.30%, respectively. Clinical parameters were compared between the two groups defined by this optimal cutoff (Table S2). High SAAR levels were significantly associated with age, LDH, FIB and PSA levels (*P* < 0.001).

### ROC curve analysis of SAAR levels in identifying bone metastasis in PCa patients

Similarly, an ROC curve was plotted for SAAR and bone metastasis (Fig. 1D). The optimal cutoff value of SAAR for identifying bone metastasis in PCa patients was determined to be 1.4150, and SAAR levels above this threshold were significantly associated with the presence of bone metastasis (*P* < 0.001). The AUC was 0.77, with a 95% CI [0.71–0.83]. Sensitivity, specificity and PPV were 68.40%, 73.10% and 75.00%, respectively. Based on this cutoff value, the patients were divided into two groups to compare clinical parameters (Table S2). High SAAR levels were significantly associated with LDH, FIB, PSA and Gleason score (*P* < 0.001).

### Incremental value of SAAR

The incremental value of SAAR was evaluated by comparing models with and without SAAR (Fig. S1). The AUC of the PSA model alone was 0.798 (95% CI [0.736–0.860]). Adding SAAR to the PSA-alone model increased the AUC from 0.798 to 0.832, although the difference was not statistically significant by the DeLong test (*P* = 0.107). Continuous NRI and IDI showed significant improvements, whereas categorical NRI did not. Furthermore, when SAAR was incorporated into the clinical baseline model (PSA + Gleason score), the AUC increased to 0.843, which was significantly higher than that of the PSA-alone model (*P* = 0.039) and the PSA + Gleason score model (*P* = 0.045). Compared with both models, the inclusion of SAAR significantly improved continuous NRI and IDI (Table S3).

### Development of the bone metastasis model

The 254 PCa patients with definitive bone metastasis status were randomly allocated to a training set (*n* = 203) and a testing set (*n* = 51). No statistically significant differences in baseline characteristics were observed between the two sets (Table S4). Within the training set, patients with bone metastasis had higher LDH, FIB, PSA and SAAR levels and a greater proportion of high pathological grade than those without bone metastasis (Table S5).

**Figure 2:**
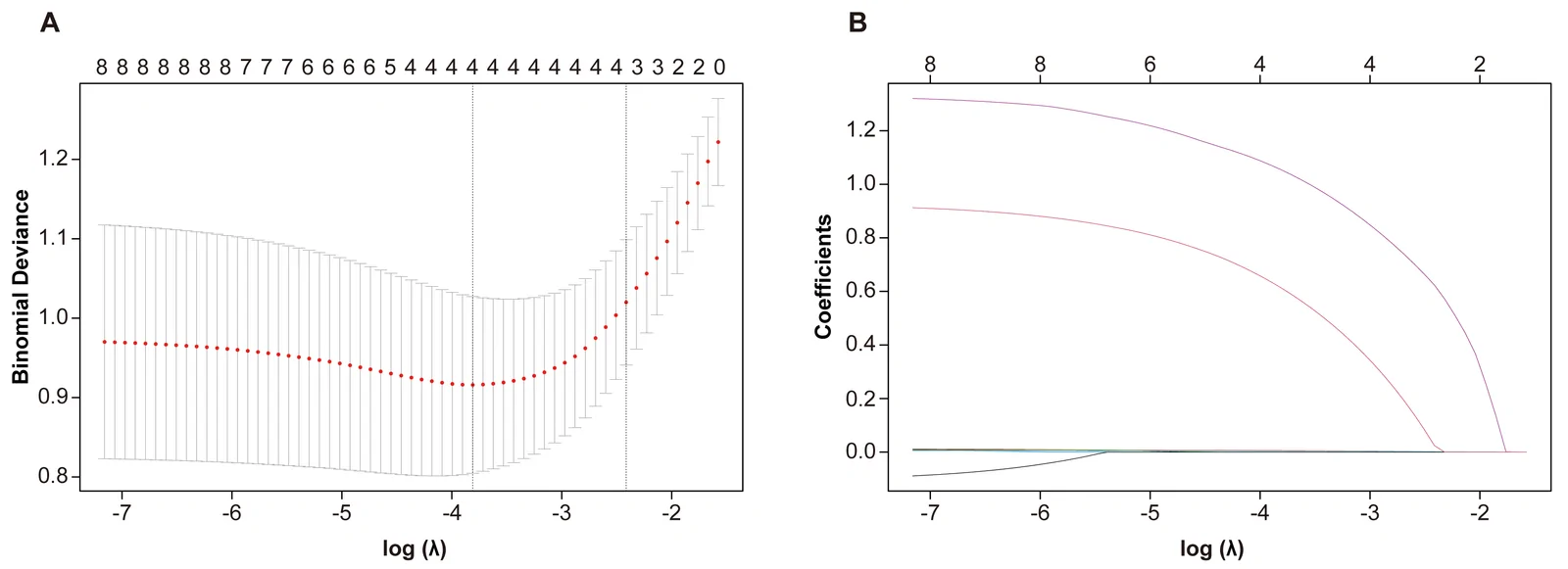
Variable selection using LASSO logistic regression for identifying bone metastasis in prostate cancer. (A) 10-fold cross-validation was performed to determine the regularization parameter (*λ*), and the curve of binomial deviance *versus* log (*λ*) was plotted. The left vertical dashed line indicates the value of *λ* that minimized the cross-validated binomial deviance (*λ*_min), whereas the right vertical dashed line indicates the largest *λ* within 1-standard error of the minimum deviance (*λ*_1se). To obtain a more parsimonious model, *λ*_1se (0.08945) was adopted for variable selection. (B) LASSO coefficient profiles of candidate variables. Each curve represents the coefficient trajectory of an individual variable. At *λ*_1se, SAAR, LDH, PSA, and pathological grade were retained for subsequent multivariable logistic regression analysis. LASSO, least absolute shrinkage and selection operator; SAAR, serum sialic acid-to-albumin ratio; LDH, lactate dehydrogenase; PSA, prostate-specific antigen. Pathological grades were classified into low-grade and high-grade groups based on the Gleason score.

Univariable logistic regression results are presented in Table S6. LASSO regression was subsequently performed in the training set using 10-fold cross-validation (Figs. 2A, 2B). At the *λ*_1se_ criterion, four variables with non-zero coefficients were retained: LDH, PSA, SAAR and pathological grade (Table S7). Following variable selection, a multivariable logistic regression model was constructed using the four retained predictors. All variables remained independently associated with the presence of bone metastasis in the multivariable logistic regression model (Table 4). Based on this model, a nomogram was constructed to visualize the probability of bone metastasis in patients with PCa (Fig. 3).

**Table 4:** Multivariable regression analysis of LASSO-selected variables for bone metastasis in the training set.

**Variables**	*β*	**S.E**	**Z**	**OR (95% CI)**	***P***
**LDH**	0.008	0.003	2.494	1.008 (1.002–1.015)	0.013
**PSA**	0.005	0.001	3.969	1.005 (1.002–1.007)	<0.001
**SAAR**	1.274	0.560	2.275	3.575 (1.193–10.713)	0.023
**Pathological grade**					
Low (≤7)				1.000 (Reference)	
High (>7)	0.927	0.423	2.192	2.526 (1.103–5.784)	0.028

**Notes.**

LDH lactate dehydrogenase PSA prostate-specific antigen SAAR serum sialic acid-to-albumin ratio OR odds ratio CI confidence interval

Pathological grades were classified into low-grade and high-grade groups based on the Gleason score.

Variables with non-zero LASSO coefficients were entered into multivariable logistic regression.

*P* < 0.05 is considered as statistically significant.

**Figure 3:**
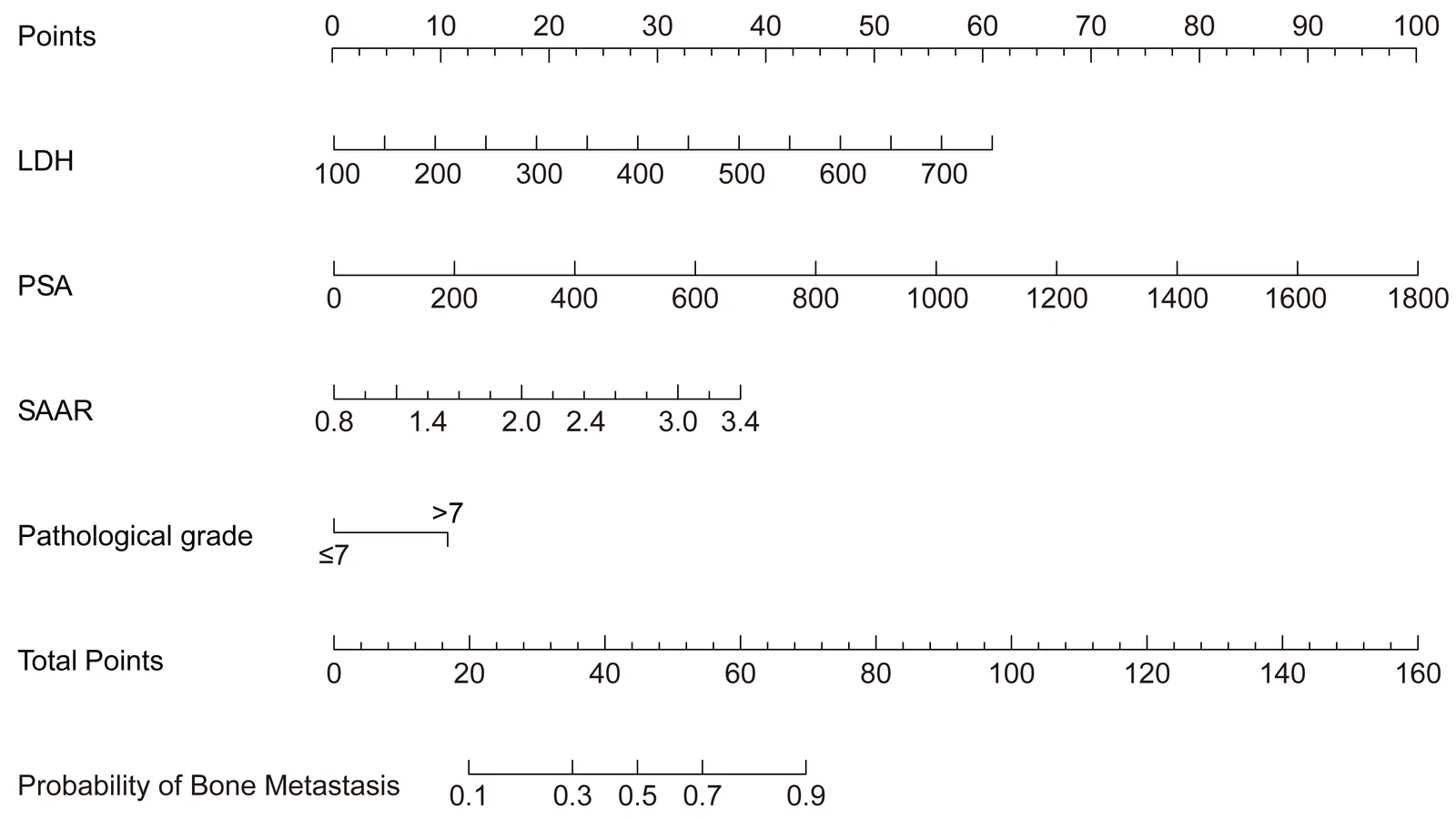
Nomogram for estimating the probability of bone metastasis in patients with prostate cancer. The nomogram incorporates SAAR, LDH, PSA, and pathological grade for estimating the probability of bone metastasis in patients with PCa. To estimate the probability of bone metastasis, locate the value of each predictor on its corresponding axis and draw a vertical line upward to the “Points” axis to determine the assigned points. Sum the points for all predictors to obtain the total points, and then project the total points vertically onto the “Probability of Bone Metastasis” axis. PCa, prostate cancer; SAAR, serum sialic acid-to-albumin ratio; LDH, lactate dehydrogenase; PSA, prostate-specific antigen. Pathological grades were classified into low-grade and high-grade groups based on the Gleason score.

### Internal validation and performance of the model

The model still retained discriminatory ability in the testing set. ROC analysis yielded an AUC of 0.860 (95% CI [0.808–0.912]) in the training set and 0.834 (95% CI [0.723–0.944]) in the testing set (Figs. 4A, 4D). The Brier scores were 0.134 and 0.163, respectively. In the training set, the model achieved an accuracy of 0.731 and a sensitivity of 0.831, whereas the corresponding values in the testing set were 0.725 and 0.850. The negative predictive values (NPV) of the model were 91% in the training set and 87% in the testing set. Detailed model evaluation metrics are provided in Table S8.

**Figure 4:**
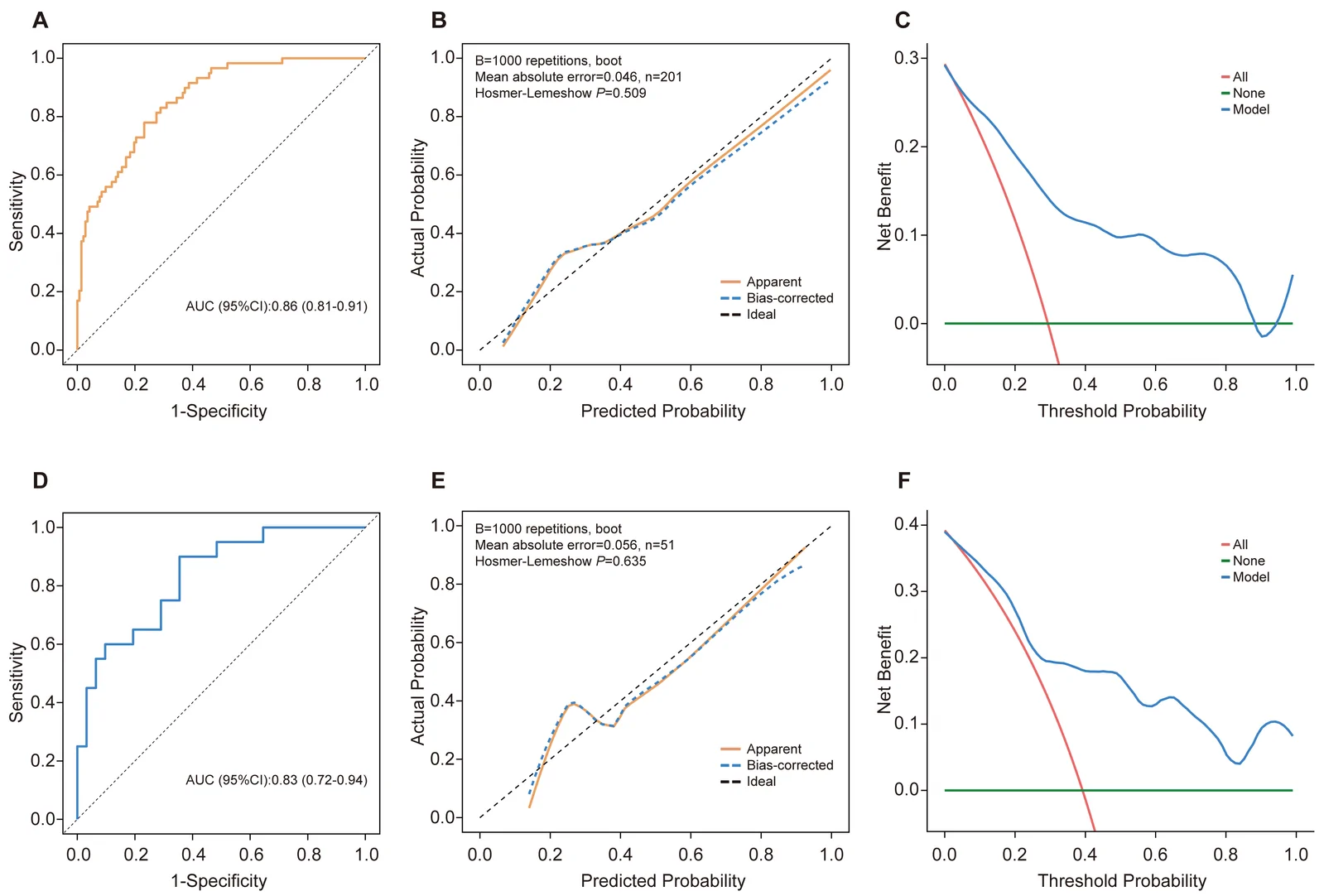
Performance of the model for identifying bone metastasis in prostate cancer patients. (A–C) Model performance in the training set. (D–F) Model performance in the testing set. (A, D) ROC curves of the model. The AUCs were 0.86 (95% CI [0.81–0.91]) in the training set and 0.83 (95% CI [0.72–0.94]) in the testing set. (B, E) Calibration curves of the model based on 1,000 bootstrap resamples. The orange solid line represents the apparent calibration, the blue dashed line represents the bootstrap bias-corrected calibration, and the black dashed line represents ideal calibration. In both the training and testing sets, the bias-corrected calibration curves were close to the ideal line, indicating good model calibration. (C, F) DCA of the model. DCA showed that the model provided a greater net benefit than both the treat-all and treat-none strategies when the threshold probability ranged from 0.05 to 0.80 in both sets. ROC, receiver operating characteristic; AUC, area under the curve; DCA, decision curve analysis.

The agreement between model estimated probabilities and observed bone metastasis status was further examined using calibration curves generated from 1,000 bootstrap resamples (Figs. 4B, 4E). The calibration intercept and slope were 0.132 and 0.858 in the testing set. No evidence of poor model fit was observed in either the training set or the testing set, as indicated by the Hosmer-Lemeshow test (*P* = 0.509 and *P* = 0.635, respectively). DCA also supported the potential clinical utility of the model. Across threshold probabilities of approximately 0.05–0.80, use of the model generally resulted in a greater net benefit than the treat-all and treat-none strategies in both sets (Figs. 4C, 4F).

## Discussion

This study retrospectively analyzed the pretreatment SAAR levels, other laboratory indicators, and clinical characteristics of 691 patients to explore the association of SAAR with PCa and bone metastasis. The results showed that SAAR was associated with PCa, although its ability to distinguish PCa from BPH was limited. In contrast, the association between SAAR and bone metastasis in PCa patients was more pronounced. SAAR provided modest incremental value beyond conventional clinical indicators. Accordingly, we developed and internally validated a model to assist in identifying bone metastasis in patients with PCa.

Currently, the primary methods for PCa screening are measuring serum PSA levels and performing digital rectal examination (DRE). Although PSA screening has significantly reduced PCa mortality to some extent (Hugosson et al., 2019; Martin et al., 2024), its low specificity has led to concerns about overdiagnosis (Ilic et al., 2018). Studies have shown that 20–50% of men diagnosed with PCa through screening may be overdiagnosed, with the overdiagnosis rate increasing with age (Pinsky & Parnes, 2023). Although prostate biopsy is used as a basis for clinical diagnosis, it carries risks, such as bleeding, infection, and urinary retention, and there is a certain rate of missed diagnoses. Liquid biopsy techniques (such as circulating tumor cells and exosomal microRNAs) have shown potential, but issues with sensitivity and standardization remain unresolved (Casanova-Salas et al., 2021; Heidrich et al., 2021; Malla et al., 2017). Recently, the use of ECT and Prostate Specific Membrane Antigen-positron emission tomography/computed tomography (PSMA-PET/CT) has improved the detection of bone metastasis (Drost et al., 2020; Jeet et al., 2023), but these methods are costly and require specialized imaging teams and equipment. Therefore, non-invasive and cost-effective serum biomarkers are needed to help identify PCa patients with a higher likelihood of bone metastasis and determine who should be prioritized for these advanced imaging examinations.

Our study found that SAAR levels were higher in PCa patients compared with BPH patients. After adjusting for other variables, SAAR levels remained independently associated with PCa. Although the standalone ability of SAAR to discriminate between PCa and BPH remains limited, its high PPV suggests that it may serve as a complementary serum parameter to PSA in supporting the clinical diagnosis of PCa. Subsequently, based on previously collected biomarkers and clinical characteristics related to PCa, patients with PCa were further grouped to study the relationship between SAAR levels and clinical pathological characteristics. SAAR levels were significantly associated with LDH, FIB, and PSA levels. Notably, SAAR was associated with pathological grade, in which patients in the high-grade group had higher levels of SAAR than those in the low-grade group. Interestingly, although the SAAR levels of advanced-stage patients were higher than those of early-stage patients, this difference did not reach statistical significance.

Given that bone metastasis is a major factor increasing complication burden and driving poor prognosis, this study focused on assessing the likelihood of bone metastasis in patients with PCa. Results showed that patients with PCa and bone metastasis had significantly higher SAAR levels than those without bone metastasis. This relationship remained significant in both univariable and multivariable logistic regression analyses, suggesting a possible independent association between SAAR and the presence of bone metastasis in PCa patients. Further comparisons across multiple models showed that adding SAAR to conventional clinical indicators modestly improved model discrimination and risk reclassification, suggesting that SAAR may provide incremental information beyond existing clinical indicators. Finally, through LASSO feature selection and internal validation, a clinical model combining SAAR, LDH, PSA, and pathological grade was developed to assist in identifying bone metastasis in PCa patients. The model demonstrated favorable discrimination in both the training and internal validation sets. The AUCs were 0.860 and 0.834, respectively, with a minimal performance drop between the two sets. Furthermore, Brier scores, calibration curves, and Hosmer-Lemeshow test results indicated good concordance between predicted probabilities and actual bone metastasis status. This model is intended for patients with newly diagnosed prostate cancer who have not yet received systemic treatment. It aims to estimate the likelihood of bone metastasis being present at initial presentation and to help identify high-risk patients who should be prioritized for further imaging evaluation, such as ECT or PSMA PET/CT.

The biological plausibility of SAAR as a composite biomarker may arise from the interplay between tumor-associated aberrant glycosylation and the systemic status of the host. SA exhibits high membrane localization in malignant cells, and serves as a key biomarker for cancer development and progression. Studies have shown that tumor cells increase SA expression to evade immune surveillance, promote metastasis, and enhance invasiveness. Consequently, SA levels are significantly elevated in various cancers, including PCa (Reily et al., 2019; Varki et al., 2009; Fuster & Esko, 2005). SA has attracted increasing attention in PCa biomarker research. Compared with BPH patients, PCa patients exhibit significantly elevated SA levels, which are strongly correlated with PSA levels (Zhang et al., 2019). By measuring serum SA levels, it is possible to assist in early PCa screening, assess tumor progression, and evaluate metastatic status. Serum SA levels may also discriminate between localized and metastatic PCa and provide a reference for monitoring the treatment efficacy. Pre-treatment ALB levels may serve as an auxiliary marker for cancer detection. Studies have demonstrated that lower ALB levels are linked to higher mortality in gastrointestinal cancers, lung cancer, kidney cancer, and gynecological tumors (Gupta & Lis, 2010). The clinical relevance of ALB in cancer has also been examined through its incorporation into composite biomarkers. For instance, the albumin-to-alkaline phosphatase ratio (AAPR) combines two routinely available laboratory parameters and has been studied as a low-cost serum biomarker. Tian et al. (2020) conducted a study involving 17 studies and 20 cohorts (5,951 patients) to evaluate the clinical application value of AAPR in the prognostic assessment of solid tumors. The results showed that higher AAPR levels were significantly associated with better tumor prognosis, making it a potentially clinically valuable biomarker for small cell lung cancer, hepatocellular carcinoma, and nasopharyngeal carcinoma. The prognostic value of preoperative albumin levels in urological tumors has also been confirmed (Ren et al., 2023). Both SA and ALB have emerged as biomarkers for PCa and bone metastasis. Elevated SA and decreased ALB levels have demonstrated significant associations with PCa (Zhang et al., 2019; Sejima et al., 2013). However, SA levels can be influenced by a variety of non-tumor factors, including acute or chronic inflammation, infection, liver dysfunction, and long-term immunosuppressant use, which may reduce its specificity in assessing PCa and bone metastasis. Nevertheless, by optimizing detection strategies and combining SA with other indicators, its clinical value may be enhanced. Notably, the current evidence lacks a critical evaluation of the potential synergistic effects when combining SA and ALB as composite biomarkers for PCa assessment. Therefore, we combined SA and ALB as SAAR to assist in identifying bone metastasis in PCa patients. By integrating elevated SA and reduced ALB levels, SAAR may more accurately reflect the metabolic and inflammatory states associated with PCa, reducing interference from single indicators. Compared with traditional biomarkers, SAAR combines multiple physiological dimensions, potentially reflecting not only tumor burden but also systemic inflammation and nutritional status, and may therefore provide a more comprehensive assessment of the disease.

To our knowledge, this is the first study to integrate SA and ALB to explore their clinical value in PCa and bone metastasis, which fills a gap in this field of research. Through analysis and validation, our findings suggest that SAAR, as a composite biomarker, may have potential for assisting in the identification of PCa and assessing the presence of bone metastasis, thereby providing useful information for clinical decision-making.

However, our study has some inherent limitations. First, its single-center retrospective cross-sectional design may have introduced selection and information biases, and the model underwent internal validation only. Therefore, external validation in multi-center prospective cohorts is warranted. Second, SA and ALB levels may be influenced by nutritional status, systemic inflammation, hepatic and renal function, and overall disease burden. Because data on C-reactive protein, detailed nutritional indicators, and complete hepatic and renal function markers were unavailable, residual confounding may remain despite the use of strict inclusion and exclusion criteria. Finally, the model is intended to assist in identifying bone metastasis rather than to establish a definitive diagnosis, and it cannot predict future bone metastasis. Future studies should incorporate additional biomarkers or imaging parameters to improve model performance and clinical applicability.

## Conclusions

SAAR was associated with PCa, although its ability to differentiate PCa from BPH was limited. In contrast, SAAR showed a stronger association with bone metastasis, and provided modest incremental value beyond conventional clinical indicators. The clinical model incorporating SAAR, LDH, PSA, and pathological grade showed favorable performance in internal validation, though external validation remains necessary.

##  Supplemental Information

10.7717/peerj.21824/supp-1 Supplemental Information 1 Raw data

10.7717/peerj.21824/supp-2 Supplemental Information 2 Data codebook

10.7717/peerj.21824/supp-3 Supplemental Information 3 Correlation between SAAR levels and clinicopathological parameters in BPH and PCa BPH: benign prostatic hyperplasia; PCa: prostate cancer; SAAR: serum sialic acid-to-albumin ratio; LDH: lactate dehydrogenase; AFU: α-L-fucosidase; FIB: fibrinogen; PSA: prostate-specific antigen; Pathological grade were classified into low-grade and high-grade groups based on the Gleason score; ECT: emission computed tomography. Skewed data are represented by the median (Q1, Q3), and categorical data are represented by frequency (percentage, %). *: Mann-Whitney U test; *P* < 0.05 is considered as statistically significant.

10.7717/peerj.21824/supp-4 Supplemental Information 4 Comparison of clinical parameters between groups according to SAAR levels (BPH vs PCa and PCa vs Bone metastasis) BPH: benign prostatic hyperplasia; PCa: prostate cancer; SAAR: serum sialic acid-to-albumin ratio; LDH: lactate dehydrogenase; AFU: α -L-fucosidase; FIB: fibrinogen; PSA: prostate-specific antigen. Pathological grade were classified into low-grade and high-grade groups based on the Gleason score. Skewed data are represented by the median (Q1, Q3), and categorical data are represented by frequency (percentage, %). *: Mann-Whitney U test; #: chi-square test; *P* < 0.05 is considered as statistically significant. Note: Missing values for smoking history, *n* = 5; §Missing values for Pathological grade, *n* = 18; ∮ Missing values for Pathological stage, *n* = 20. Missing values were excluded from the corresponding statistical tests, and percentages were calculated based on available data.

10.7717/peerj.21824/supp-5 Supplemental Information 5 Incremental value of SAAR for bone metastasis risk in PCa patients SAAR, serum sialic acid-to-albumin ratio; PSA, prostate-specific antigen; AUC, area under the receiver operating characteristic curve; CI, confidence interval; IDI, integrated discrimination improvement; NRI, net reclassification improvement.

10.7717/peerj.21824/supp-6 Supplemental Information 6 Baseline clinical characteristics of patients in the training and testing sets LDH: lactate dehydrogenase; AFU: α -L-fucosidase; FIB: fibrinogen; PSA: prostate-specific antigen; SAAR: serum sialic acid-to-albumin ratio. Pathological grade were classified into low-grade and high-grade groups based on the Gleason score. Skewed data are represented by the median (Q1, Q3), and categorical data are represented by frequency (percentage, %). *: Mann-Whitney U test; #: chi-square test; *P* < 0.05 is considered as statistically significant. Note: Missing value for smoking history, *n* = 1, from the train group; §missing values for Pathological grade, *n* = 2, from the train group. Missing values were excluded from the corresponding statistical tests, and percentages were calculated based on available data.

10.7717/peerj.21824/supp-7 Supplemental Information 7 Baseline clinical characteristics of PCa patients with and without bone metastasis in the training set PCa: prostate cancer; LDH: lactate dehydrogenase; AFU: α -L-fucosidase; FIB: fibrinogen; PSA: prostate-specific antigen; SAAR: serum sialic acid-to-albumin ratio. Pathological grades were classified into low-grade and high-grade groups based on the Gleason score. Skewed data are represented by the median (Q1, Q3), and categorical data are represented by frequency (percentage, %). *: Mann-Whitney U test; #: chi-square test; *P* < 0.05 is considered as statistically significant. Note: Missing value for smoking history, *n* = 1, from the non-bone metastasis group; §missing values for Pathological grade, *n* = 2, from the non-bone metastasis group. Missing values were excluded from the corresponding statistical tests, and percentages were calculated based on available data.

10.7717/peerj.21824/supp-8 Supplemental Information 8 Univariable regression analysis of variables associated with bone metastasis in the training set LDH, lactate dehydrogenase; AFU, α -L-fucosidase; FIB, fibrinogen; PSA, prostate-specific antigen; SAAR, serum sialic acid-to-albumin ratio. Pathological grades were classified into low-grade and high-grade groups based on the Gleason score. OR, odds ratio; CI, confidence interval; *P* < 0.05 is considered as statistically significant.

10.7717/peerj.21824/supp-9 Supplemental Information 9 Variables selected by LASSO logistic regression in the training group LDH, lactate dehydrogenase; AFU, α -L-fucosidase; FIB, fibrinogen; PSA, prostate-specific antigen; SAAR, serum sialic acid-to-albumin ratio. Pathological grades were classified into low-grade and high-grade groups based on the Gleason score. Variables with non-zero coefficients were selected for subsequent multivariable logistic regression analysis.

10.7717/peerj.21824/supp-10 Supplemental Information 10 Predictive performance of the model in the training and testing sets AUC, area under the receiver operating characteristic curve; PPV, positive predictive value; NPV, negative predictive value; CI, confidence interval.

10.7717/peerj.21824/supp-11 Supplemental Information 11 Incremental value of SAAR for identifying bone metastasis in prostate cancer patients (A) ROC curves for the individual predictors (Gleason score, PSA, and SAAR) and their combined models. (B) ROC curves for multiple models after adjustment for selected covariates. Comparisons across multiple models suggested that adding SAAR to conventional clinical indicators modestly improved model discrimination. ROC, receiver operating characteristic; SAAR, serum sialic acid-to-albumin ratio.

10.7717/peerj.21824/supp-12 Supplemental Information 12 STROBE checklist

## Additional Information and Declarations

### Data Availability

The following information was supplied regarding data availability: The raw data are available in the Supplemental Files.
