# Supplementary material for: Association of the serum sialic acid-to-albumin ratio with prostate cancer and bone metastasis: a retrospective cross-sectional study
Source: PeerJ. 2026 Oct 27;14:e21824. doi: 10.7717/peerj.21824 (PMC13721882; doi:10.7717/peerj.21824)
Supplement: Supplementary file 1 [file peerj-14-21824-s011.pdf]

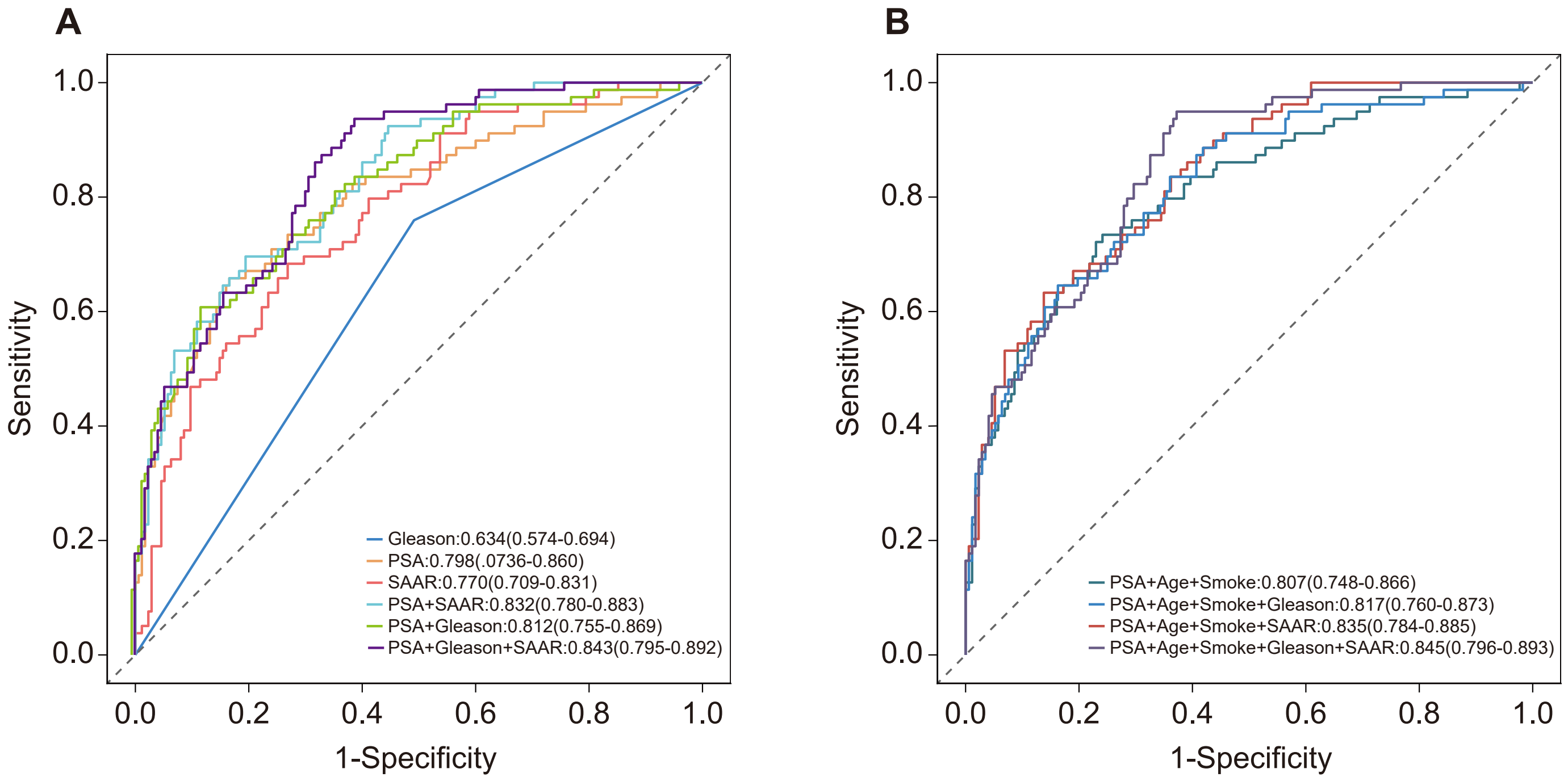
A
B
Sensitivity
1-Specificity
0.0
0.2
0.4
0.6
0.8
1.0
Gleason:0.634(0.574-0.694)
PSA:0.798(.0736-0.860)
SAAR:0.770(0.709-0.831)
PSA+SAAR:0.832(0.780-0.883)
PSA+Gleason:0.812(0.755-0.869)
PSA+Gleason+SAAR:0.843(0.795-0.892)
PSA+Age+Smoke:0.807(0.748-0.866)
PSA+Age+Smoke+Gleason:0.817(0.760-0.873)
PSA+Age+Smoke+SAAR:0.835(0.784-0.885)
PSA+Age+Smoke+Gleason+SAAR:0.845(0.796-0.893)
