# Supplementary material for: Association of the serum sialic acid-to-albumin ratio with prostate cancer and bone metastasis: a retrospective cross-sectional study
Source: PeerJ. 2026 Oct 27;14:e21824. doi: 10.7717/peerj.21824 (PMC13721882; doi:10.7717/peerj.21824)
Supplement: Supplementary file 2 [file peerj-14-21824-s003.docx]

**Table S1 Correlation between SAAR levels and clinicopathological parameters in BPH and PCa.**

| **Parameters** | **BPH (n=185)** | **SAAR levels** | ***P*** | **PCa (n=506)** | **SAAR levels** | ***P*** |
| --- | --- | --- | --- | --- | --- | --- |
| **Age** | ≤68 (n=94) | 1.21 (1.14, 1.34) | 0.310^*^ | ≤70 (n=260) | 1.30 (1.15, 1.60) | 0.150^*^ |
|  | >68 (n=91) | 1.26 (1.15, 1.43) |  | >70 (n=246) | 1.34 (1.20, 1.57) |  |
| **Smoking history** | Yes (n=63) | 1.24 (1.13, 1.43) | 0.308^*^ | Yes (n=186) | 1.40 (1.22, 1.68) | **<0.001**^*^ |
|  | No (n=122) | 1.20 (1.15, 1.38) |  | No (n=315) | 1.29 (1.16, 1.53) |  |
|  |  |  |  | Missing (n=5) | - |  |
| **LDH** | ≤188 (n=94) | 1.20 (1.13, 1.35) | **0.038**^*^ | ≤194 (n=260) | 1.28 (1.15, 1.48) | **<0.001**^*^ |
|  | >188 (n=91) | 1.26 (1.15, 1.43) |  | >194 (n=246) | 1.41 (1.21, 1.68) |  |
| **AFU** | ≤17 (n=104) | 1.24 (1.15, 1.43) | 0.552^*^ | ≤17 (n=279) | 1.33 (1.18, 1.62) | 0.709^*^ |
|  | >17 (n=81) | 1.21 (1.13, 1.38) |  | >17 (n=227) | 1.32 (1.17, 1.55) |  |
| **FIB** | ≤2.91 (n=93) | 1.19 (1.10, 1.26) | **<0.001**^*^ | ≤3.19 (n=254) | 1.21 (1.10, 1.32) | **<0.001**^*^ |
|  | >2.91 (n=92) | 1.34 (1.17, 1.51) |  | >3.19 (n=252) | 1.55 (1.33, 1.84) |  |
| **PSA** | ≤5.74 (n=93) | 1.23 (1.14, 1.35) | 0.464^*^ | ≤40.75 (n=253) | 1.26 (1.14, 1.45) | **<0.001**^*^ |
|  | >5.74 (n=92) | 1.22 (1.13, 1.43) |  | >40.75 (n=253) | 1.41 (1.23, 1.72) |  |
| **Pathological grade** | - | - | - | Low (GS ≤7, n=215) | 1.29 (1.15, 1.50) | **0.001**^*^ |
|  | - | - | - | High (GS >7, n=273) | 1.37 (1.20, 1.65) |  |

**Table S1 (Continued)**

| **Parameters** | **BPH (n=185)** | **SAAR levels** | ***P*** | **PCa (n=506)** | **SAAR levels** | ***P*** |
| --- | --- | --- | --- | --- | --- | --- |
|  |  |  |  | Missing (n=18) | - |  |
| **Pathological T stage** | - | - | - | pT1-2 (n=357) | 1.32 (1.16, 1.57) | 0.319^*^ |
|  | - | - | - | pT3-4 (n=129) | 1.34 (1.19, 1.62) |  |
|  |  |  |  | Missing (n=20) | - |  |
| **ECT** | - | - | - | Without bone metastasis (n=175) | 1.26 (1.12, 1.44) | **<0.001**^*^ |
|  | - | - | - | With bone metastases (n=79) | 1.59 (1.34, 1.93) |  |
|  |  |  |  | Suspicion (n=92) | 1.35 (1.25, 1.71) |  |
|  |  |  |  | Not performed (n=160) | 1.28 (1.16, 1.54) |  |

BPH: benign prostatic hyperplasia; PCa: prostate cancer; SAAR: serum sialic acid-to-albumin ratio; LDH: lactate dehydrogenase; AFU: α-L-fucosidase; FIB: fibrinogen; PSA: prostate-specific antigen; Pathological grade were classified into low-grade and high-grade groups based on the Gleason score; ECT: emission computed tomography. Skewed data are represented by the median (Q1, Q3), and categorical data are represented by frequency (percentage, %). *: Mann-Whitney U test; *P* < 0.05 is considered as statistically significant.
