# Supplementary material for: Association of the serum sialic acid-to-albumin ratio with prostate cancer and bone metastasis: a retrospective cross-sectional study
Source: PeerJ. 2026 Oct 27;14:e21824. doi: 10.7717/peerj.21824 (PMC13721882; doi:10.7717/peerj.21824)
Supplement: Supplementary file 3 [file peerj-14-21824-s004.docx]

**Table S2 Comparison of clinical parameters between groups according to SAAR levels (BPH vs PCa and PCa vs Bone metastasis).**

| **Parameters** | **SAAR ≤1.2958**  **(n=348)** | **SAAR >1.2958 (n=343)** | ***P*** | **SAAR ≤1.4150**  **(n=310)** | **SAAR >1.4150 (n=196)** | ***P*** |
| --- | --- | --- | --- | --- | --- | --- |
| **Age** | 69.00 (62.25, 74.00) | 71.00 (65.00, 77.00) | **<0.001**^*^ | 70.00 (64.00, 75.00) | 70.50 (65.00, 77.00) | 0.253^*^ |
| **LDH** | 187.00(168.00, 208.00) | 198.00(176.00, 238.00) | **<0.001**^*^ | 188.00(170.75, 208.25) | 211.00(180.00, 263.00) | **<0.001**^*^ |
| **AFU** | 17.00 (13.00, 21.00) | 16.00 (13.00, 22.00) | 0.414^*^ | 17.00 (13.00, 22.00) | 16.00 (12.25, 21.00) | 0.161^*^ |
| **FIB** | 2.74 (2.51, 3.07) | 3.56 (3.15, 4.17) | **<0.001**^*^ | 2.87 (2.59, 3.25) | 3.83 (3.36, 4.56) | **<0.001**^*^ |
| **PSA** | 11.70 (6.30, 41.92) | 37.5 (10.10, 151.50) | **<0.001**^*^ | 27.09 (10.88, 71.83) | 94.92 (21.73, 276.93) | **<0.001**^*^ |
| **Smoking history**^✟^  (no vs yes) | 234/110 | 203/139 | **0.018^#^** | 208/97 | 107/89 | **0.002^#^** |
| **Pathological grade**^§^  (GS ≤7 vs >7) | - | - | - | 148/152 | 67/121 | **0.003^#^** |
| **Pathological stage**^∮^  (pT1-2 vs pT3-4) | - | - | - | 225/76 | 132/53 | 0.415**^#^** |
| **PCa patients** | 227 (65.2%) | 279 (81.3%) | **<0.001^#^** | - | - | - |
| **Bone metastasis patients** | - | - | - | 25 (16.34%) | 54 (53.5%) | **<0.001^#^** |

BPH: benign prostatic hyperplasia; PCa: prostate cancer; SAAR: serum sialic acid-to-albumin ratio; LDH: lactate dehydrogenase; AFU: α-L-fucosidase; FIB: fibrinogen; PSA: prostate-specific antigen. Pathological grade were classified into low-grade and high-grade groups based on the Gleason score. Skewed data are represented by the median (Q1, Q3), and categorical data are represented by frequency (percentage, %). *: Mann-Whitney U test; #: chi-square test; *P* < 0.05 is considered as statistically significant.

Note: ✟ Missing values for smoking history, n = 5; § Missing values for Pathological grade, n = 18; ∮ Missing values for Pathological stage, n = 20. Missing values were excluded from the corresponding statistical tests, and percentages were calculated based on available data.
