# Supplementary material for: Association of the serum sialic acid-to-albumin ratio with prostate cancer and bone metastasis: a retrospective cross-sectional study
Source: PeerJ. 2026 Oct 27;14:e21824. doi: 10.7717/peerj.21824 (PMC13721882; doi:10.7717/peerj.21824)
Supplement: Supplementary file 4 [file peerj-14-21824-s005.docx]

**Table S3** **Incremental value of SAAR for bone metastasis risk in PCa patients.**

| **Model** | **AUC** | **Delong test** | **Categorical NRI**  **(95% CI)** | ***P*** | **Continuous NRI**  **(95% CI)** | ***P*** | **IDI**  **(95% CI)** | ***P*** |
| --- | --- | --- | --- | --- | --- | --- | --- | --- |
| **PSA** | 0.798 |  |  |  |  |  |  |  |
| **SAAR** | 0.770 | 0.484 | -0.122 (-0.277-0.033) | 0.123 | -0.057 (-0.321-0.208) | 0.675 | -0.085 (-0.159--0.012) | **0.023** |
| **PSA+SAAR** | 0.832 | 0.107 | 0.001 (-0.093-0.095) | 0.980 | 0.535 (0.280-0.790) | **<0.001** | 0.052 (0.020-0.084) | **0.002** |
| **PSA+Gleason+SAAR** | 0.843 | **0.039** | 0.156 (0.015-0.296) | **0.030** | 0.556 (0.299-0.813) | **<0.001** | 0.069 (0.033-0.106) | **<0.001** |
| **PSA+Gleason** | 0.812 |  |  |  |  |  |  |  |
| **PSA+Gleason+SAAR** | 0.843 | **0.045** | 0.087 (-0.019-0.193) | 0.109 | 0.450 (0.191-0.710) | **<0.001** | 0.038 (0.010-0.065) | **0.007** |
| **PSA+Age+Smoke** | 0.807 |  |  |  |  |  |  |  |
| **PSA+Age+Smoke+SAAR** | 0.835 | 0.184 | 0.001 (-0.122-0.125) | 0.985 | 0.412 (0.154-0.671) | **0.002** | 0.051 (0.019-0.083) | **0.002** |
| **PSA+Age+Smoke+Gleason** | 0.817 |  |  |  |  |  |  |  |
| **PSA+Age+Smoke+Gleason+SAAR** | 0.845 | **0.076** | 0.144 (0.039-0.250) | **0.007** | 0.385 (0.124-0.645) | **0.004** | 0.036 (0.009-0.064) | **0.010** |

SAAR: serum sialic acid-to-albumin ratio; PSA: prostate-specific antigen; AUC: area under the receiver operating characteristic curve; CI: confidence interval;

IDI: integrated discrimination improvement; NRI: net reclassification improvement.
