# Supplementary material for: Association of the serum sialic acid-to-albumin ratio with prostate cancer and bone metastasis: a retrospective cross-sectional study
Source: PeerJ. 2026 Oct 27;14:e21824. doi: 10.7717/peerj.21824 (PMC13721882; doi:10.7717/peerj.21824)
Supplement: Supplementary file 5 [file peerj-14-21824-s006.docx]

**Table S4 Baseline clinical characteristics of patients in the training and testing sets.**

| **Variables** | **Total (n = 254)** | **Train (n = 203)** | **Test (n = 51)** | ***P*** |
| --- | --- | --- | --- | --- |
| **Age** | 70.00 (64.00, 75.00) | 70.00 (64.00, 75.00) | 70.00 (64.00, 76.50) | 0.833^*^ |
| **LDH** | 194.00 (173.00, 224.00) | 193.00 (171.00, 224.00) | 196.00 (179.50, 223.50) | 0.365^*^ |
| **AFU** | 17.00 (14.00, 23.00) | 17.00 (13.50, 23.00) | 19.00 (14.50, 23.00) | 0.305^*^ |
| **FIB** | 3.15 (2.74, 3.64) | 3.10 (2.72, 3.62) | 3.27 (2.92, 3.67) | 0.136^*^ |
| **PSA** | 54.38 (16.13, 173.01) | 49.21 (14.96, 145.70) | 76.04 (25.39, 219.80) | 0.104^*^ |
| **SAAR** | 1.34 (1.17, 1.60) | 1.33 (1.17, 1.58) | 1.40 (1.20, 1.67) | 0.349^*^ |
| **Smoking history**^✟^ |  |  |  | 0.147^#^ |
| No | 161 (63.64) | 133 (65.84) | 28 (54.90) |  |
| Yes | 92 (36.36) | 69 (34.16) | 23 (45.10) |  |
| **Pathological grade**^§^ |  |  |  | 0.400^#^ |
| Low (≤7) | 107 (42.46) | 88 (43.78) | 19 (37.25) |  |
| High (>7) | 145 (57.54) | 113 (56.22) | 32 (62.75) |  |
| **Clinical outcome** |  |  |  | 0.161^#^ |
| Non-bone metastasis | 175 (68.90) | 144 (70.94) | 31 (60.78) |  |
| Bone metastasis | 79 (31.10) | 59 (29.06) | 20 (39.22) |  |

LDH: lactate dehydrogenase; AFU: α-L-fucosidase; FIB: fibrinogen; PSA: prostate-specific antigen; SAAR: serum sialic acid-to-albumin ratio. Pathological grade were classified into low-grade and high-grade groups based on the Gleason score. Skewed data are represented by the median (Q1, Q3), and categorical data are represented by frequency (percentage, %). *: Mann-Whitney U test; #: chi-square test; *P* < 0.05 is considered as statistically significant.

Note: ✟ Missing value for smoking history, n = 1, from the train group; § missing values for Pathological grade, n = 2, from the train group. Missing values were excluded from the corresponding statistical tests, and percentages were calculated based on available data.
