# Supplementary material for: Association of the serum sialic acid-to-albumin ratio with prostate cancer and bone metastasis: a retrospective cross-sectional study
Source: PeerJ. 2026 Oct 27;14:e21824. doi: 10.7717/peerj.21824 (PMC13721882; doi:10.7717/peerj.21824)
Supplement: Supplementary file 6 [file peerj-14-21824-s007.docx]

**Table S5 Baseline clinical characteristics of PCa patients with and without bone metastasis** **in the training set.**

| **Variables** | **Total (n = 203)** | **Non-bone metastasis (n = 144)** | **Bone metastasis (n = 59)** | ***P*** |
| --- | --- | --- | --- | --- |
| **Age** | 70.00 (64.00, 75.00) | 70.00 (64.00, 75.00) | 69.00 (65.50, 76.00) | 0.761^*^ |
| **LDH** | 193.00 (171.00, 224.00) | 186.00 (168.00, 212.00) | 212.00 (180.00, 266.00) | **<0.001**^*^ |
| **AFU** | 17.00 (13.50, 23.00) | 16.50 (13.00, 22.00) | 19.00 (15.00, 24.00) | 0.186^*^ |
| **FIB** | 3.10 (2.72, 3.62) | 2.98 (2.64, 3.40) | 3.61 (2.90, 4.36) | **<0.001**^*^ |
| **PSA** | 49.21 (14.96, 145.70) | 30.95 (11.55, 73.52) | 200.30 (64.64, 463.49) | **<0.001**^*^ |
| **SAAR** | 1.33 (1.17, 1.58) | 1.26 (1.13, 1.44) | 1.55 (1.33, 1.93) | **<0.001**^*^ |
| **Smoking history**^✟^ |  |  |  | 0.353^#^ |
| No | 133 (65.84) | 97 (67.83) | 36 (61.02) |  |
| Yes | 69 (34.16) | 46 (32.17) | 23 (38.98) |  |
| **Pathological grade**^§^ |  |  |  | **<0.001**^#^ |
| Low (≤7) | 88 (43.78) | 74 (52.11) | 14 (23.73) |  |
| High (>7) | 113 (56.22) | 68 (47.89) | 45 (76.27) |  |

PCa: prostate cancer; LDH: lactate dehydrogenase; AFU: α-L-fucosidase; FIB: fibrinogen; PSA: prostate-specific antigen; SAAR: serum sialic acid-to-albumin ratio. Pathological grades were classified into low-grade and high-grade groups based on the Gleason score. Skewed data are represented by the median (Q1, Q3), and categorical data are represented by frequency (percentage, %). *: Mann-Whitney U test; #: chi-square test; *P* < 0.05 is considered as statistically significant.

Note: ✟ Missing value for smoking history, n = 1, from the non-bone metastasis group; § missing values for Pathological grade, n = 2, from the non-bone metastasis group. Missing values were excluded from the corresponding statistical tests, and percentages were calculated based on available data.
