# Supplementary material for: Association of the serum sialic acid-to-albumin ratio with prostate cancer and bone metastasis: a retrospective cross-sectional study
Source: PeerJ. 2026 Oct 27;14:e21824. doi: 10.7717/peerj.21824 (PMC13721882; doi:10.7717/peerj.21824)
Supplement: Supplementary file 7 [file peerj-14-21824-s008.docx]

**Table S6** **Univariable regression analysis of variables associated with bone metastasis in the training set.**

| **Variables** | **β** | **S.E** | **Z** | **OR (95%CI)** | ***P*** |
| --- | --- | --- | --- | --- | --- |
| **Age** | 0.005 | 0.020 | 0.244 | 1.005 (0.966-1.045) | 0.807 |
| **LDH** | 0.012 | 0.003 | 3.782 | 1.012 (1.006-1.019) | **<0.001** |
| **AFU** | 0.020 | 0.022 | 0.920 | 1.020 (0.977-1.066) | 0.358 |
| **FIB** | 0.407 | 0.143 | 2.850 | 1.503 (1.136-1.989) | **0.004** |
| **PSA** | 0.006 | 0.001 | 4.866 | 1.006 (1.004-1.009) | **<0.001** |
| **SAAR** | 2.421 | 0.496 | 4.882 | 11.262 (4.260-29.774) | **<0.001** |
| **Smoking history** |  |  |  |  |  |
| No |  |  |  | 1.000 (Reference) |  |
| Yes | 0.298 | 0.321 | 0.927 | 1.347 (0.718-2.529) | 0.354 |
| **Pathological grade** |  |  |  |  |  |
| Low (≤7) |  |  |  | 1.000 (Reference) |  |
| High (>7) | 1.252 | 0.349 | 3.587 | 3.498 (1.765-6.934) | **<0.001** |

LDH: lactate dehydrogenase; AFU: α-L-fucosidase; FIB: fibrinogen; PSA: prostate-specific antigen; SAAR: serum sialic acid-to-albumin ratio. Pathological grades were classified into low-grade and high-grade groups based on the Gleason score. OR: odds ratio; CI: confidence interval; *P* < 0.05 is considered as statistically significant.
