# Supplementary material for: Association of the serum sialic acid-to-albumin ratio with prostate cancer and bone metastasis: a retrospective cross-sectional study
Source: PeerJ. 2026 Oct 27;14:e21824. doi: 10.7717/peerj.21824 (PMC13721882; doi:10.7717/peerj.21824)
Supplement: Supplementary file 8 [file peerj-14-21824-s009.docx]

**Table S7** **Variables selected by LASSO logistic regression in the training group.**

| **Variables** | **LASSO coefficient (β)** | **Selected** |
| --- | --- | --- |
| **Age** | 0.0000 | No |
| **LDH** | **0.0017** | Yes |
| **AFU** | 0.0000 | No |
| **FIB** | 0.0000 | No |
| **PSA** | **0.0015** | Yes |
| **SAAR** | **0.6238** | Yes |
| **Smoking history** | 0.0000 | No |
| **Pathological grade** | **0.0243** | Yes |

LDH: lactate dehydrogenase; AFU: α-L-fucosidase; FIB: fibrinogen; PSA: prostate-specific antigen; SAAR: serum sialic acid-to-albumin ratio. Pathological grades were classified into low-grade and high-grade groups based on the Gleason score. Variables with non-zero coefficients were selected for subsequent multivariable logistic regression analysis.
