# Supplementary material for: Association of the serum sialic acid-to-albumin ratio with prostate cancer and bone metastasis: a retrospective cross-sectional study
Source: PeerJ. 2026 Oct 27;14:e21824. doi: 10.7717/peerj.21824 (PMC13721882; doi:10.7717/peerj.21824)
Supplement: Supplementary file 9 [file peerj-14-21824-s010.docx]

**Table S8** **Predictive performance of the model in the training and testing sets.**

| **Model** | **AUC (95%CI)** | **Accuracy (95%CI)** | **Sensitivity (95%CI)** | **Specificity (95%CI)** | **PPV (95%CI)** | **NPV (95%CI)** | **F1 score** | **Brier score** |
| --- | --- | --- | --- | --- | --- | --- | --- | --- |
| **Train** | 0.860 (0.808-0.912) | 0.731 (0.664-0.791) | 0.831 (0.735-0.926) | 0.690 (0.614-0.766) | 0.527 (0.425-0.628) | 0.907 (0.853-0.962) | 0.645 | 0.134 |
| **Test** | 0.834 (0.723-0.944) | 0.725 (0.583-0.841) | 0.850 (0.694-1.000) | 0.645 (0.477-0.814) | 0.607 (0.426-0.788) | 0.870 (0.732-1.000) | 0.708 | 0.163 |

AUC: area under the receiver operating characteristic curve; PPV: positive predictive value; NPV: negative predictive value; CI: confidence interval.
